# Epidemiological dynamics and molecular characterization of HIV drug resistance in eastern China from 2020 to 2023

**DOI:** 10.3389/fmicb.2024.1475548

**Published:** 2024-10-18

**Authors:** Min Zhu, Zhou Sun, Xingliang Zhang, Wenjie Luo, Sisheng Wu, Ling Ye, Ke Xu, Junfang Chen

**Affiliations:** Department of HIV/AIDS Control and Prevention, Hangzhou Center for Disease Control and Prevention (Hangzhou Health Supervision Institution), Hangzhou, China

**Keywords:** HIV drug resistance, molecular network, Bayesian analysis, molecular epidemiology, transmitted drug resistance

## Abstract

**Objective:**

HIV drug resistance (HIVDR) has become a threat to the elimination of the AIDS epidemic due to the global scale-up of antiretroviral therapy (ART) for HIV-infected individuals. This study aims to investigate the epidemiological dynamics and molecular characterization of HIV pretreatment drug resistance (PDR) and acquired drug resistance (ADR) in Hangzhou, a developed region in China.

**Methods:**

An epidemiological survey combined with a molecular transmission network and Bayesian analysis was conducted. A total of 3,596 individuals with newly confirmed HIV infections (from 2020 to 2023) and 164 individuals with ART failure (from 2021 to 2023) were included. The molecular transmission network was used to identify key drug-resistant transmission clusters, while the Bayesian analysis was utilized to trace the origins and spread of these clusters.

**Results:**

The overall prevalence of PDR was found to be 8.4% (303/3596). Among these cases, PDR to non-nucleoside reverse transcriptase inhibitors (NNRTIs) accounted for 4.7% (170/3596), significantly higher than the resistance observed for protease inhibitors (PIs; 2.8%, *p* < 0.001) and nucleoside reverse transcriptase inhibitors (NRTIs; 1.4%, *p* < 0.001). Multivariate logistic regression analysis revealed a significantly higher PDR value among individuals infected with the CRF07_BC subtype compared to those with the CRF08_BC subtype (aOR = 0.56, 95% CI = 0.359–0.859, *p* = 0.008). The molecular transmission network analysis identified the transmission of the drug resistance mutation (DRM) Q58E within the clusters of the CRF07_BC subtype. The Bayesian analysis suggested that these clusters were introduced into Hangzhou from Shenzhen between 2005 and 2012. Furthermore, the study highlighted 50.6% (83/164) prevalence of ADR among individuals experiencing ART failure. The combined molecular network analysis of virological failure and newly confirmed HIV infections indicated the transmission of the K103N mutation between these groups.

**Conclusion:**

In conclusion, targeted interventions may be necessary for specific subtypes and transmission clusters to control the spread of drug-resistant HIV. Continuous monitoring of resistance patterns is critical to inform treatment strategies and optimize ART regimens.

## 1 Introduction

In 2016, the World Health Organization (WHO) recommended that all individuals diagnosed with HIV should immediately begin antiretroviral therapy (ART) to significantly reduce new HIV infections. However, the widespread use of antiretroviral drugs is likely to increase the prevalence of acquired drug resistance (ADR) in treated individuals and transmitted drug resistance (TDR) in newly infected individuals (Phillips et al., 2014; Clutter et al., 2016).

HIV pretreatment drug resistance (PDR) is detected in individuals who are either antiretroviral drug-naive people or have prior exposure to antiretroviral drugs and are initiating or reinitiating first-line ART. This includes cases of transmitted drug resistance (TDR) as well as individuals resuming therapy after prior treatment (WHO, 2017a). The WHO’s report on HIV drug resistance highlighted that an increasing number of countries have reached the 10% threshold for PDR to non-nucleoside reverse transcriptase inhibitors (NNRTIs; WHO, 2021). The WHO recommends that when this threshold is reached in a surveyed country, urgent changes to the first-line HIV treatment regimen should be implemented (WHO, 2017a).

In China, the overall prevalence of PDR remains modest at 7.4% (Chen et al., 2023). However, certain provinces exhibit higher prevalence rates, such as Shanghai (17.4%; Wang et al., 2019), Tianjin (11.5%; Zeng et al., 2020), Chongqing (10.5%; Liu et al., 2022), Shenyang (9.1%; Zhao et al., 2021), Jilin (9.2%), and Zhejiang (8.8%; Chen et al., 2023). Therefore, timely monitoring of drug resistance mutations (DRMs) is crucial for providing a scientific basis for large-scale prevention and control programs.

Molecular transmission networks and phylogenetic analysis, such as Bayesian phylogenetic tree analysis based on HIV gene sequence data, are valuable tools for defining HIV transmission clusters and tracking the dynamics of HIV transmission. Bayesian methods, by incorporating prior knowledge and accounting for uncertainties in the evolutionary process, provide robust statistical frameworks for estimating the timing and direction of transmissions. This allows researchers to reconstruct transmission pathways more accurately over time. By combining drug resistance analysis with molecular transmission networks, Bayesian phylogenetic methods enable a more precise identification of resistant strain clusters, which helps inform public health strategies aimed at controlling HIV transmission (Zhao et al., 2021; Xu et al., 2021; Zheng et al., 2022).

Hangzhou, the capital of Zhejiang Province and a central city in the Yangtze River Delta region, is known for its thriving digital economy and e-commerce. By 2020, it had a rapidly growing population of 11.9 million long-term residents (Hangzhou Bureau of Statistics, 2023), including a floating population of 5.0 million, which accounts for 42.0% of the long-term residents (Zhejiang Bureau of Statistics, 2022). The large migrant population increases the risk of HIV transmission. Studies have indicated that the prevalence of HIV in Hangzhou is diverse and that relatively high prevalence of TDR in new infections has been found in Hangzhou. However, comprehensive data on PDR and ADR in Hangzhou in recent years are limited. In this study, we conducted a large-scale survey to assess the level of PDR and ADR. We constructed a molecular transmission network to explore drug resistance-related transmission. Furthermore, Bayesian analysis was conducted to trace the transmission clusters. Characterizing the risk and source of HIV drug resistance enables timely and effective interventions to prevent the spread of resistant strains.

## 2 Materials and methods

### 2.1 Study population and data collection

In this study, 3,596 individuals with newly confirmed HIV infections from 2020 to 2023 and 164 individuals with ART failure from 2021 to 2023 were enrolled from Hangzhou, Zhejiang Province, China. Blood samples and socio-demographic characteristics, including sex, age, ethnicity, education, current place of residence, marital status, infection route, and high-risk sexual behavior, were collected. All participants provided informed consent, and the study was approved by the Medical Ethics Committee of the Hangzhou Municipal Center for Disease Control and Prevention.

### 2.2 HIV nucleic acid extraction, amplification, and sequencing

Plasma was separated from whole blood at 3,000 × g for 10 min and then stored at −80°C. HIV-1 RNA was extracted from the plasma using the QIAamp Viral RNA Mini Kit (QIAGEN, Germany). The pol gene fragments (1,316 bp, HXB2: 2147–3,462) were amplified using reverse transcription polymerase chain reaction (RT–PCR) and nested polymerase chain reaction (PCR). The amplification products from the PCR-positive samples were sent to Hangzhou TsingKe Bio-Tech Co. for purification and sequencing. The obtained sequence fragments were assembled using Sequencher 5.4.6 (Gene Codes, Ann Arbor, MI, United States).

### 2.3 HIV subtype analysis and drug resistance analysis

The sequences obtained were aligned with the HIV reference sequences downloaded from the Los Alamos National Laboratory HIV Sequence Database and corrected using MEGA v11.0.13. Subtypes were identified using HIV BLAST.^1^ The sequences were then uploaded to the Stanford HIV Drug Resistance Database^2^ for the analysis of resistance mutation sites.

### 2.4 Construction of the HIV-1 molecular transmission network

The HIV-1 molecular transmission network was established using the HIV-TRACE algorithm (Kosakovsky Pond et al., 2018). Pairwise genetic distances of the sequences were calculated with the TN93 model using HyPhy (Pond et al., 2005). The HIV molecular transmission network was constructed with a 0.5% distance threshold, which is recommended by the CDC in the United States and China (CDC, 2018; Oster et al., 2018). The network was visualized in Cytoscape v3.10.1.

### 2.5 Bayesian evolutionary analysis

Forty-four CRF07_BC reference sequences from other regions in China were downloaded from GenBank based on their high homology (>98.0%) with the participants in drug-resistant transmission clusters. BEAST v.1.10.4 under an uncorrelated relaxed clock model, the GTR + G + I nucleotide substitution model, and a Bayesian skyline plot demographic model were used to perform Bayesian evolutionary analysis (Suchard et al., 2018). The BEAST analysis was performed using Markov Chain Monte Carlo (MCMC) runs of 100 million generations, with samples taken every 10,000 steps. The Bayesian MCMC output was analyzed using Tracer v1.7.2 (Rambaut et al., 2018). Maximum clade credibility (MCC) trees were generated using TreeAnnotator v1.10.4 and visually edited in FigTree v1.4.4.

### 2.6 Statistical analysis

A chi-squared test, Fisher’s exact test, and two-way ANOVA were performed in GraphPad Prism 9. Turkey multiple comparisons test was performed after the two-way ANOVA. The Chi-square test was employed as univariable regression. Multivariable logistic regression was performed in SPSS 25. The odds ratios (OR), adjusted odds ratios (aOR), and 95% confidence intervals (CI) were calculated.

## 3 Results

### 3.1 Demographic characteristics of the study population

Out of 4,595 plasma samples from newly confirmed HIV infections and 211 plasma samples from individuals with ART failure, 78.2% (3,596/4595) and 77.8% (164/211) were successfully sequenced, respectively. The participant counts of newly confirmed HIV infections and individuals with ART failure in the following analyzed datasets were N1 = 3,596 and N2 = 164, respectively (Table 1). There was no significant difference between the participants and non-participants in our analysis.

**Table 1:** Demographic characteristics of the newly reported HIV infections and HIV infections with ART failure in this study.

Variable	Newly reported HIV infections number (%)	HIV infections with ART failure number (%)
Total	3,596 (100.0)	164 (100.0)
Sex		
Male	3,254 (90.5)	149 (90.9)
Female	342 (9.5)	15 (9.1)
Age		
Older people (≥50 years old)	792 (22.0)	37 (22.6)
Younger people (< 50 years old)	2,804 (78.0)	127 (77.4)
Ethnicity		
Han	3,426 (95.3)	159 (97.0)
Others	170 (4.7)	5 (3.0)
Education		
Illiterate	70 (1.9)	6 (3.7)
Primary school	450 (12.5)	20 (12.2)
Junior high school	859 (23.9)	39 (23.8)
Senior high school and over	2,217 (61.7)	99 (60.4)
Marital status		
Unmarried	2053 (57.1)	93 (56.7)
Married	922 (25.6)	40 (24.4)
Divorced or widowed	575 (16.0)	30 (18.3)
Unknown	46 (1.3)	1 (0.6)
Infection route		
Homosexual route	2,327 (65.7)	91 (55.5)
Heterosexual route	1,134 (31.5)	69 (42.1)
Others and unknown	135 (3.8)	4 (2.4)
Subtype		
CRF07_BC	1,648 (45.8)	/
CRF01_AE	1,219 (33.9)	/
CRF08_BC	188 (5.2)	/
CRF55_01B	159 (4.4)	/
B	82 (2.3)	/
URF(CRF07_BC/CRF01_AE)	111 (3.1)	/
Others	189 (5.3)	/
With DR		
Yes	303 (8.4)	83 (50.6)
No	3,293 (91.6)	81 (49.4)
CD4 count before ART (cells/μL)		
<200	1,145 (31.8)	/
200–500	1913 (53.2)	/
>500	358 (10.0)	/
Unknown	180 (5.0)	/
Viral load (copies/ml)		
1,000–10,000	/	59 (36.0)
> 10,000	/	105 (64.0)

Among the newly confirmed HIV infections, the majority were male (90.5%, 3,254/3,596), younger individuals (78.0%, 2,804/3,596), and of Han ethnicity (95.3%, 3,426/3,596). Over half had at least a high school education (61.7%, 2,217/3,596), were unmarried (57.1%, 2,053/3,596), acquired HIV through homosexual contact (65.7%, 2,327/3,596), and had a CD4+ T cell count between 200 and 500 cells/μL (53.2%, 1,913/3,596). The most common HIV subtype was CRF07_BC (45.8%, 1,648/3,596), followed by CRF01_AE (33.9%, 1,219/3,596), CRF08_BC (5.2%, 188/3,596), CRF55_01B (4.4%, 159/3,596), URF (CRF07_BC/CRF01_AE; 3.1%, 111/3,596), and the subtype B (2.3%, 82/3,596). The individuals with ART failure exhibited similar proportions in terms of sex, age, ethnicity, education level, marital status, and infection route.

### 3.2 Drug resistance analysis

The overall prevalence of PDR among the newly confirmed HIV infections was 8.4% (303/3596; Table 2). Resistance to NNRTIs accounted for 4.7% (170/3596), which was significantly higher than resistance to protease inhibitors (PIs; 2.8%, *p* < 0.001) and nucleoside reverse transcriptase inhibitors (NRTIs; 1.4%, *p* < 0.001). Among NNRTI agents, the highest proportion of PDR was observed for nevirapine (NVP; 3.5%, 127/3596) and efavirenz (EFV; 3.4%, 121/3596), followed by RPV (2.8%, 101/3596), DOR (1.1%, 38/3596), and ETR (1.1%, 38/3596). For NRTI agents, the proportion of PDR for ABC, AZT, D4T, DDI, FTC, 3TC, and TDF was 0.7% (24/3596), 0.5% (17/3596), 0.5% (17/3596), 0.8% (30/3596), 0.4% (14/3596), 0.6% (21/3596), 0.6% (21/3596), and 0.2% (8/3596), respectively. Among PI agents, the proportion of PDR was mainly to TPV/r (1.8%, 65/3598) and NFV (0.9%, 33/3596), with PDR to other PI agents being low, around 0.1%. Notably, resistance to three or more NNRTI agents was more common (1.9%, 69/3596) than resistance to three or more NRTI agents (0.7%, *p* < 0.001) or PI agents (0.1%, *p* < 0.001). The most frequent drug resistance mutations were Q58E (1.9%, 67/3596), K103N/R/S (1.7%, 62/3596), and E138A/G/K (1.5%, 55/3596).

**Table 2:** Drug resistance and mutations of the newly reported HIV infections in this study.

Antiretroviral Drug	Number	% (N1 = 3,596)	HIV Drug Resistance Mutations (n, %)
Total	303	8.4	
PIs	101	2.8	Q58E (67, 1.9), M46I/L (20, 0.6), L10F (3, 0.1), N88S (2, 0.1), K20T (2, 0.1), T74P (2, 0.1), others (6, 0.2)
ATV/r	4	0.1	
FPV/r	6	0.2	
IDV/r	5	0.1	
LPV/r	3	0.1	
NFV	33	0.9	
SQV/r	4	0.1	
TPV/r	65	1.8	
Drug resistance ≥3	5	0.1	
NRTIs	49	1.4	M184I/V (17, 0.5), T215A/D/N/S/I (10, 0.3), M41L (6, 0.2), D67N/G (7, 0.2), K65R/N (4, 0.1), K70R/T/N (5, 0.1), L210W (2, 0.1), others (2, 0.1)
ABC	24	0.7	
AZT	17	0.5	
D4T	30	0.8	
DDI	14	0.4	
FTC	21	0.6	
3TC	21	0.6	
TDF	8	0.2	
Drug resistance ≥3	26	0.7	
NNRTIs	170	4.7	K103N/R/S (62, 1.7), E138A/G/K (55, 1.5), V179I/L/D (26, 0.7), V106I/M (16, 0.4), K101E/Q (13, 0.4), G190E/A/S (10, 0.3), A98G (6, 0.2), V108I (4, 0.1), others (4, 0.1)
DOR	38	1.1	
EFV	121	3.4	
ETR	38	1.1	
NVP	127	3.5	
RPV	101	2.8	
Drug resistance ≥3	69	1.9	

Factors associated with HIV PDR among the newly confirmed HIV infections are listed in Table 3. Univariable and multi-variable logistic regression analyses showed a statistically significant association between the HIV subtype and PDR. After adjusting for other factors, the individuals with the CRF07_BC subtype were more likely to cluster in transmission networks than those with the CRF08_BC subtype (aOR = 0.56, 95% CI = 0.359–0.859, *p* = 0.008).

**Table 3:** Factors associated with HIV PDR among the newly confirmed HIV infections.

Variable	Newly confirmed HIV infections number	Drug resistance number (%)	Univariable analysis		Multivariable logistic regression	
			c^2	*p*-value	aOR (95% CI)	*p-*value
Age			2.27	0.518		
10–29	1,521	116 (7.6)			1	
30–39	790	70 (8.9)			0.879 (0.623–1.24)	0.462
40–49	493	46 (9.3)			0.899 (0.558–1.449)	0.662
≥ 50	792	71 (9.0)			1.007 (0.611–1.658)	0.979
Sex			1.55	0.214		
Male	3,254	269 (8.3)				
Female	342	35 (10.2)				
Marital status			4.62	0.202		
Unmarried	2053	167 (8.1)			1	
Married	922	77 (8.4)			1.356 (0.899–2.046)	0.146
Divorced or widowed	575	58 (10.1)			1.045 (0.682–1.601)	0.841
Unknown	46	1 (2.2)			4.976 (0.66–37.525)	0.12
Education			4.30	0.038		
0 year	70	5 (7.1)			1	
1–6 years	450	43 (9.6)			0.66 (0.249–1.749)	0.404
7–9 years	859	92 (10.7)			0.585 (0.223–1.534)	0.275
> 9 years	2,217	163 (7.4)			0.832 (0.312–2.216)	0.713
Infection route			6.36	0.042		
Homosexual route	2,327	177 (7.6)			1	
Heterosexual route	1,134	115 (10.1)			0.808 (0.588–1.111)	0.19
Others and unknown	135	11 (8.1)			0.766 (0.386–1.518)	0.445
Subtype			25.04	0.0003		
CRF07_BC	1,648	146 (8.9)			1	
CRF01_AE	1,219	85 (7.0)			1.297 (0.982–1.713)	0.067
CRF08_BC	188	28 (14.9)			0.555 (0.359–0.859)	0.008
CRF55_01B	159	14 (8.8)			1.007 (0.567–1.788)	0.982
B	82	11 (13.4)			0.627 (0.325–1.211)	0.165
URF(CRF07_BC/CRF01_AE)	111	1 (0.9)			10.692 (1.482–77.146)	0.019
Others	189	18 (9.5)			0.923 (0.552–1.545)	0.762
CD4 count before ART (cells/μL)			0.32	0.852		
<200	1,145	106 (9.3)			1	
200–500	1913	166 (8.7)			0.9 (0.682–1.186)	0.454
>500	358	31 (8.7)			0.899 (0.581–1.39)	0.632
Unknown	180	0 (0)			1.329 (0.695–2.544)	0.39

aOR, adjusted odds ratios; CI, confidence intervals.

The overall prevalence of ADR among the individuals with ART failure was 50.6% (83/164; Table 4). Similar to PDR, ADR to NNRTIs (46.3%, 76/164) was significantly higher than that to NRTIs (31.7%, *p* < 0.05) and PIs (3.7%, *p* < 0.001). The predominant drug resistance mutations observed were M184I/V (29.9%, 49/164), K103N (21.3%, 35/164), V179E (13.4%, 22/164), and V106I/M (9.8%, 16/164). Among the 164 individuals with ART failure, eight had viral loads exceeding 1,000 copies/mL for three consecutive years. We analyzed the dynamic changes in drug resistance mutations under drug selection pressure over these 3 years. In the first year, five NRTI resistance mutations and four NNRTI resistance mutations were detected. In the second year, one additional NRTI mutation was observed, bringing the total to six, and two additional NNRTI mutations were found, also bringing the total to six. By the third year, the number of the NRTI resistance mutations remained at six, while the NNRTI mutations increased to nine, with three new mutations added. No PI resistance mutations were detected during these 3 years. Among the NRTI resistance mutations, M184V/I (58.8%, 10/17) showed the highest mutation frequency, while among the NNRTI mutations, K103N (26.3%, 5/19), V106M (15.8%, 3/19), and G190A (15.8%, 3/19) were the most frequent. These results suggest that with prolonged antiretroviral treatment, drug resistance mutations accumulate over time, ultimately leading to clinical drug resistance.

**Table 4:** Drug resistance and mutations of the individuals with ART failure in this study.

Antiretroviral Drug	Number	% (N2 = 164)	HIV drug resistance mutations (n, %)
Total	83	50.6	
PIs	6	3.7	Q58E (5, 3.0), M46L (1, 0.6)
NFV	1	0.6	
TPV/r	5	3.0	
NRTIs	52	31.7	M184I/V (49, 29.9), T215Y/*F* (10, 6.1), M41L (9, 5.5), D67N (9, 5.5), K65R (13, 7.9), K70R/T/E/G/Q (12, 7.3), Y115F (7, 4.3), L74I/V (3, 1.8), L210W (2,1.2)
ABC	52	31.7	
AZT	12	7.3	
D4T	28	17.1	
DDI	35	21.3	
FTC	52	31.7	
3TC	52	31.7	
TDF	25	15.2	
Drug resistance ≥3	52	31.7	
NNRTIs	76	46.3	K103N (35,21.3), E138A/G/Q (3, 1.8), V179E (22, 13.4), V106I/M (16, 9.8), K101E/Q/H/P/T (11, 6.7), G190A/S (12, 7.3), A98G (3, 1.8), V108I (6, 3.7), P225H (5, 3.0), Y181C (7, 4.3), F227L (9, 5.5), Y188L/C (6, 3.7)
DOR	37	22.6	
EFV	73	44.5	
ETR	22	13.4	
NVP	73	44.5	
RPV	32	19.5	
Drug resistance ≥3	43	26.2	

### 3.3 HIV molecular transmission network analysis

**Figure 1:**
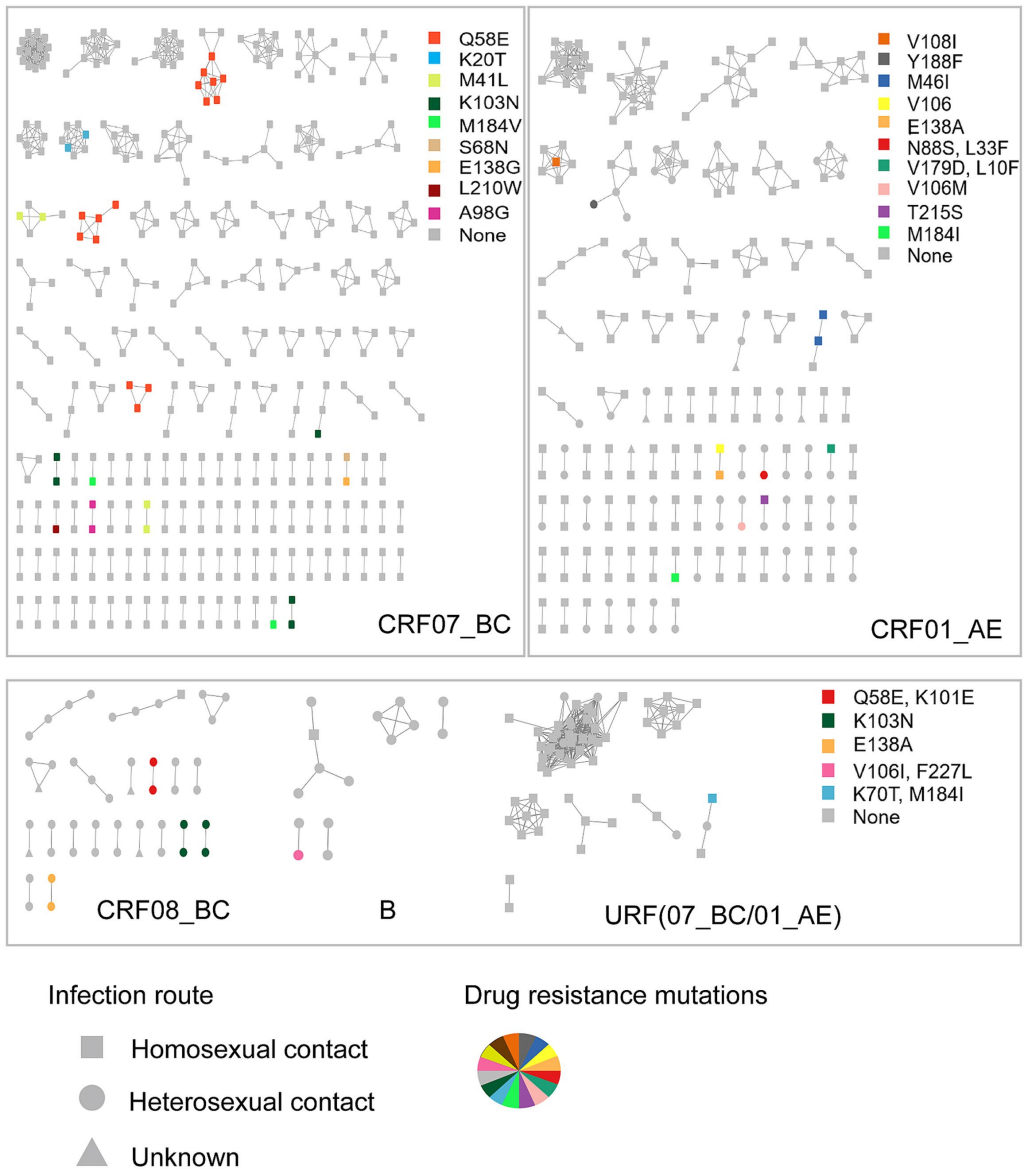
The molecular network of the newly reported HIV infections in Hangzhou. Sample links with a pairwise genetic distance <0.5% were chosen to construct the network. The clusters were partitioned according to the subtypes. Gray nodes represent the individuals with no PDR. Colored nodes represent different PDR mutations. The shape square, circle, and triangle represent the route of HIV infection through homosexual contact, heterosexual contact, and unknown, respectively.

To further investigate the characteristics of HIV drug resistance transmission, we constructed a molecular transmission network using links with a genetic distance <0.5% (Figure 1). A total of 861 individuals (23.9%, 861/3596) with 1,372 links were clustered in 278 clusters, ranging in size from 2 to 35, with 103 clusters containing three or more individuals. Among these, 54 infections with PDR were observed in 29 clusters, 10 of which included three or more individuals. We defined clusters with ≥3 nodes as large clusters.

The individuals with PDR were predominantly distributed among those infected with the CRF07_BC subtype (61.1%, 33/54), which was significantly higher than those infected with CRF01_AE (20.4%, 11/54), CRF08_BC (14.8%, 8/54), CRF55_01B (0, 0/54), URF (CRF07_BC/CRF01_AE; 1.9%, 1/54), and subtype B (1.9%, 1/54). Notably, the individuals with PDR distributed among those with CRF07_BC were more frequently found in the large clusters (60.0%, 6/10). The primary drug resistance mutations within the CRF07_BC clusters were Q58E (45.5%, 15/33), followed by K103N (15.2%, 5/33), M41L (12.1%, 4/33), and K20T (6.0%, 2/33). Fifteen individuals carrying the Q58E mutation were distributed among three clusters. The transmission fitness of the Q58E mutant strain was calculated by dividing the clustering rate of the mutant strain by the clustering rate of the wild-type strain. The transmission fitness of the Q58E mutant strain was found to be 1.02 [(15/60) / (808/3,293)].

In the CRF01_AE clusters, two individuals carried the M46I mutation, while one individual had the V108I mutation and another had the Y188F mutation; all were part of the large clusters. The individuals infected with the CRF08_BC subtype were generally older, with an average age of 51 years, and 87.5% were infected through heterosexual contact. Drug resistance mutations in the CRF08_BC clusters, including Q58E, K101E, K103N, and E138A, were all found in smaller clusters with two individuals each.

**Figure 2:**
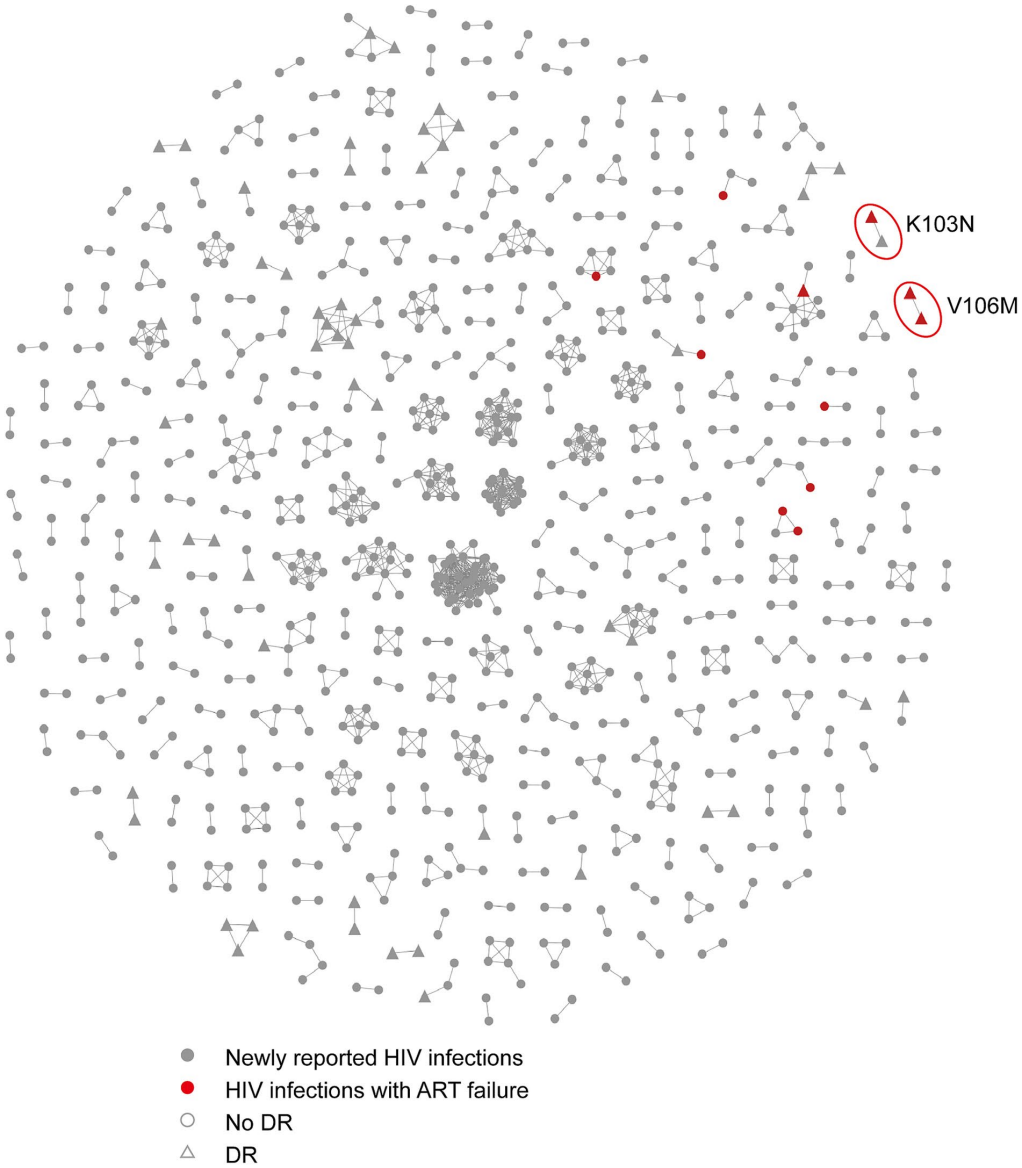
The molecular network combining the HIV infections with ART failure and newly confirmed HIV infections in Hangzhou. Sample links with a pairwise genetic distance <0.5% were chosen to construct the network. The red circles represent the clusters associated with ART failures that include resistance mutations. Gray nodes and red nodes represent the newly reported HIV infections and HIV infections with ART failure, respectively. Colored nodes represent different PDR mutations. The circle and triangle represent the individuals with no DR and with DR, respectively.

To assess the impact of ART on HIV transmission in Hangzhou, we expanded the molecular network to include both newly confirmed HIV infections and infections with ART failure (Figure 2). A total of 11 individuals with ART failure were clustered in the network. Nine of these individuals were linked to newly confirmed infections within eight clusters, and two individuals were connected to each other in one additional cluster. Among those with ART failure, 75.0% had a viral load exceeding 10,000 copies/mL compared to 25.0% with a viral load below 10,000 copies/mL. Four clusters were associated with ART failure, including one cluster that contained both newly confirmed infections and individuals with ART failure, all of whom carried the K103N drug resistance mutation.

### 3.4 Bayesian evolutionary analysis of the drug-resistant transmission clusters

Through the above HIV molecular transmission network analysis, 15 individuals carrying Q58E were identified within the drug-resistant transmission clusters of the CRF07_BC subtype. The clusters were named Q58E-C1, Q58E-C2, and Q58E-C3. The majority in the Q58E-C1 cluster were students (88.9%, 8/9), with an average age of 19. The majority of the individuals in the Q58E-C2 cluster lived in the Gongshu district (80.0%, 4/5). All individuals in these three clusters were men who have sex with men (MSM).

**Figure 3:**
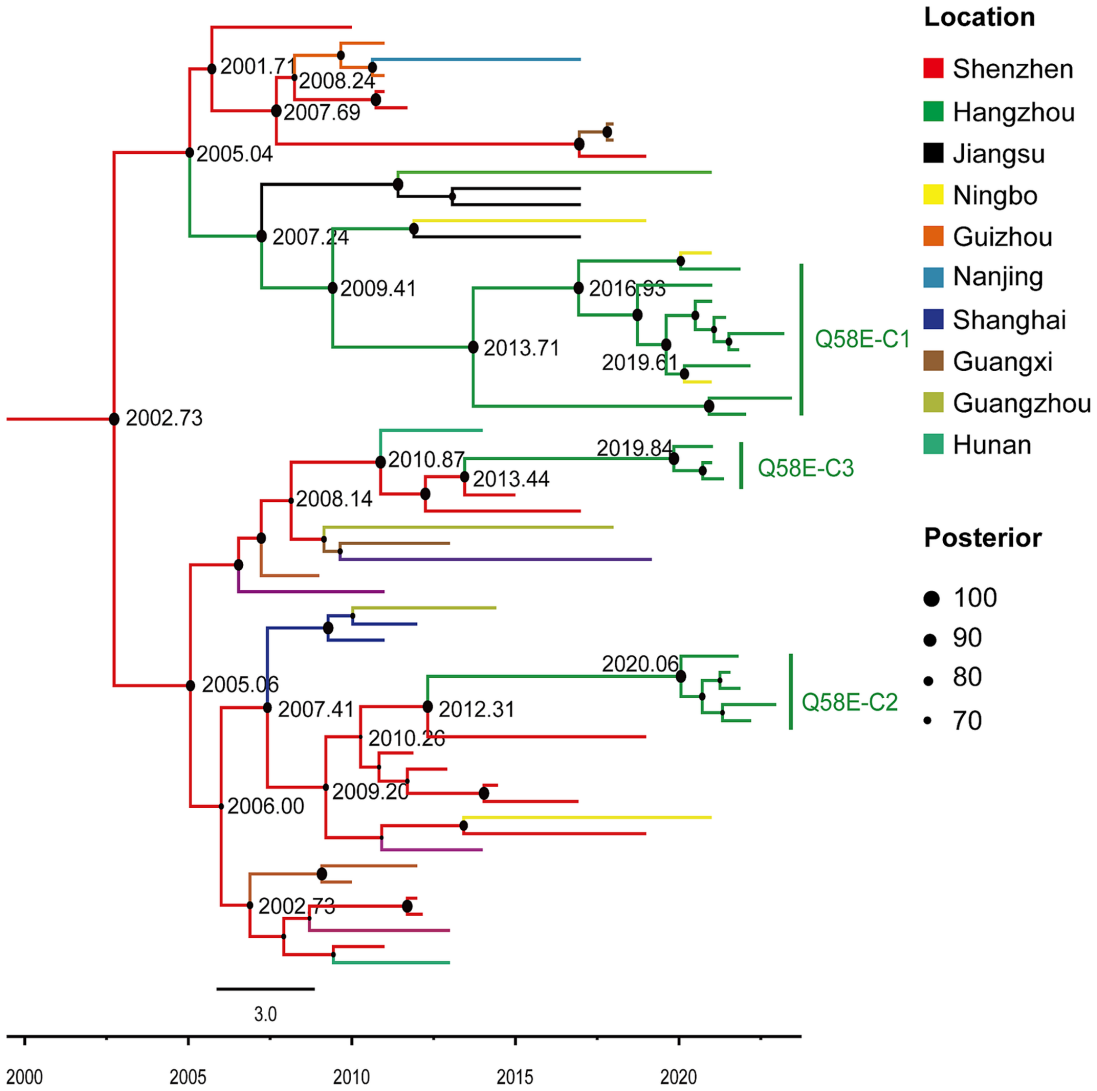
Maximum clade credibility (MCC) tree with the information of the sample location and evolutionary time analyzed by BEAST v1.10.4 and constructed by FigTree v1.4.4. In this study, in the MCC tree, 74 sequences of the CRF07_BC subtype were included, among which 59 reference sequences from cities outside of Hangzhou in China and 15 sequences from clusters Q58E-C1, Q58E-C2, and Q58E-C3. Different colors of the branches represent different source locations of the reference sequences. The branch lengths represent the evolutionary time, and nodes labeled with the evolutionary time are supported by a high posterior probability (≥ 70). The corresponding time scale is marked at the bottom of the MCC tree.

To investigate the origins of the Q58E-C1, Q58E-C2, and Q58E-C3 drug-resistant transmission clusters, Bayesian analysis was conducted to reconstruct the epidemic history of these clusters (Figure 3). We used TempEst v1.5.3 to test the molecular clock hypothesis of the selected 59 sequences. The results showed that the calculated R2 was 0.6272. The GTR model, a Log normal relaxed clock model, and a Bayesian skyline demographic growth model were used to estimate the origin time of the most recent common ancestor of the drug-resistant transmission clusters. The Skyline plot result revealed that the drug-resistant transmission clusters had an exponential growth trend from 2005 to 2010, which stabilized from 2010 to 2017 and declined in 2018. The average evolution rate of the clusters was 9.71 × 10–4 nucleotide substitutions/site/year (95% HPD: 6.70 × 10–4-1.28 × 10–3). With the support of high posterior probability, the Q58E-C1, Q58E-C2, and Q58E-C3 clusters were all introduced from Shenzhen, among which Q58E-C2 and Q58E-C3 were introduced around 2010 and showed a stable local epidemic, while the Q58E-C1 cluster was introduced around 2005 and subsequently transmitted among Hangzhou, Jiangsu, and Ningbo.

## 4 Discussion

In this study, we investigated the characteristics of HIV PDR and ADR in Hangzhou using a large sample of patients with newly confirmed HIV infections and those experiencing ART failure. The overall prevalence of PDR in Hangzhou was 8.4%, which is classified as moderate (5–15%) according to the WHO (2019). This prevalence is higher than the national average of 7.4% reported in China (Chen et al., 2023) and exceeds the prevalence observed in other regions, such as Guangxi (7.2%; Pang et al., 2021), Hefei (6.4%; Zheng et al., 2022), and Beijing (6.7%; Li et al., 2022).

The prevalence of PDR to NNRTIs (4.7%) was significantly higher than that for PIs (2.8%) and NRTIs (1.4%), which aligns with the trends observed across China. The high prevalence of PDR to NNRTIs may be related to their widespread use as first-line regimens in China since 2016 (General Office of the National Health and Family Planning Commission, 2016). The primary resistance mutations associated with NNRTIs in our study were K103N/R/S, E138A/G/K, and V179I/L/D, which target drugs such as efavirenz (EFV) and nevirapine (NVP). These findings are consistent with a nationwide survey of individuals with HIV/AIDS who had not yet initiated ART (Kang et al., 2020). Importantly, NNRTIs are known for their high rate of cross-resistance and overall strong resistance to treatment.

For NRTIs, the main mutations observed were M184I/V, which target drugs such as ABC, FTC, and 3TC. The WHO’s recommended first-line therapy includes TDF, AZT +3TC + EFV, or NVP (WHO, 2017b). The M184V/I mutation, selected by exposure to FTC and 3TC, significantly reduces the efficacy of these drugs, underscoring the importance of preventing the spread of these resistant strains.

PDR can be influenced by a range of complex factors. Our multivariate analysis indicated that the CRF07_BC subtype is more likely to harbor pretreatment drug resistance. Since CRF07_BC is the predominant HIV-1 subtype in China, targeted strategies are necessary to curb the transmission of drug-resistant strains within this subtype.

The HIV-1 molecular transmission network is a valuable tool for examining the connections between individuals in different risk groups. Clusters with three or more individuals are generally considered to carry a higher risk of onward HIV transmission. Our molecular network analysis identified individuals with drug resistance mutations Q58E, K20T, M41L, K103N, V108I, and Y188F within the key clusters of the CRF07_BC and CRF01_AE subtypes. Of particular importance is the Q58E mutation found within the CRF07_BC transmission clusters, which suggests that this mutation has relatively high viral fitness, allowing it to spread effectively even in the absence of drug pressure. Viral fitness refers to the ability of a virus to replicate and propagate within its host’s environment. Resistance mutations often confer a replication advantage in drug-exposed settings but may incur a fitness cost in drug-free environments (Stekler et al., 2018). However, in the case of the Q58E mutation, its fitness level (measured at 1.02) is comparable to that of the wild-type virus, indicating that it imposes little or no fitness cost. This allows Q58E-containing viruses to form stable transmission clusters, similar to wild-type strains. Previous studies, such as the one by Wertheim, have shown that other mutations such as K103N, Y181C, and L90M also exhibit transmission fitness comparable to or even greater than that of wild-type strains, demonstrating their ability to establish and persist in large transmission networks, even among untreated individuals (Wertheim et al., 2017). Given the high fitness of Q58E, it is plausible that this mutation could contribute to long-standing transmission chains, highlighting the need for vigilant monitoring and containment strategies.

Moreover, mutations such as K103N and Y188F, which confer high-level resistance to EFV and NVP, both of which are commonly included in free ART regimens in China (Zuo et al., 2020), underscore the importance of targeted interventions to control the spread of resistant strains within high-risk clusters. Enhanced surveillance and targeted prevention and treatment strategies are crucial for mitigating the impact of these resistant mutations.

The prevalence of ADR among the individuals with ART failure was 50.6%, which is comparable to the national average in China (51.33%; Yuan et al., 2022). Among the individuals with ADR, the most common resistance mutations were M184I/V, K103N, and V179E. Our molecular network analysis, which included both patients with newly confirmed HIV infections and those with ART failure, revealed that the K103N mutation may have been transmitted between these groups.

This study has some limitations. First-generation sequencing can only detect the dominant strain in individuals infected with HIV, resulting in an underestimation of PDR. Future studies should employ next-generation sequencing to identify a wider range of drug-resistant variants. In addition, the analysis of the pol region did not include all drug resistance mutation sites. For individuals in key clusters, it would be beneficial to analyze full-length sequences in future research.

## 5 Conclusion

The high prevalence of both pretreatment drug resistance (PDR) and acquired drug resistance (ADR) observed in Hangzhou highlights the ongoing challenges posed by ART implementation. The findings reveal significant subtype-specific differences in PDR, with the CRF07_BC-associated resistance mutations, such as Q58E, transmitting within the transmission clusters. This suggests that targeted interventions may be necessary for specific subtypes and transmission clusters. The introduction of these resistant strains from other regions, as suggested by the Bayesian analysis, emphasizes the need for robust surveillance and preventive measures to control the spread of drug-resistant HIV. In addition, the observed transmission of the resistance mutations, such as K103N, between the newly infected individuals and those with ART failure indicates a critical need for continuous monitoring of resistance patterns to inform treatment strategies. Overall, this study provides valuable insights into the epidemiology and transmission dynamics of HIVDR, which are essential for developing effective public health policies and optimizing ART regimens to minimize the impact of drug-resistant HIV strains.

## Data Availability

The datasets presented in this study can be found in online repositories. The names of the repository/repositories and accession number(s) can be found at: https://www.ncbi.nlm.nih.gov/genbank/, OR834988-OR835133, PQ218031-PQ218079.

## Footnotes

^1^https://www.hiv.lanl.gov/content/sequence/BASIC_BLAST/basic_blast.html

^2^https://hivdb.stanford.edu
